# A Prospective Analysis of Airborne Metal Exposures and Risk of Parkinson Disease in the Nurses’ Health Study Cohort

**DOI:** 10.1289/ehp.1307218

**Published:** 2014-06-06

**Authors:** Natalia Palacios, Kathryn Fitzgerald, Andrea L. Roberts, Jaime E. Hart, Marc G. Weisskopf, Michael A. Schwarzschild, Alberto Ascherio, Francine Laden

**Affiliations:** 1 Department of Nutrition, Harvard School of Public Health, Boston, Massachusetts, USA; 2 Channing Division of Network Medicine, Department of Medicine, Brigham and Women’s Hospital and Harvard Medical School, Boston, Massachusetts, USA; 3 Department of Epidemiology, Harvard School of Public Health, Boston, Massachusetts, USA; 4 Department of Neurology, Massachusetts General Hospital, Boston, Massachusetts, USA

## Abstract

Background: Exposure to metals has been implicated in the pathogenesis of Parkinson disease (PD).

Objectives: We sought to examine in a large prospective study of female nurses whether exposure to airborne metals was associated with risk of PD.

Methods: We linked the U.S. Environmental Protection Agency (EPA)’s Air Toxics tract-level data with the Nurses’ Health Study, a prospective cohort of female nurses. Over the course of 18 years of follow-up from 1990 through 2008, we identified 425 incident cases of PD. We examined the association of risk of PD with the following metals that were part of the first U.S. EPA collections in 1990, 1996, and 1999: arsenic, antimony, cadmium, chromium, lead, manganese, mercury, and nickel. To estimate hazard ratios (HRs) and 95% CIs, we used the Cox proportional hazards model, adjusting for age, smoking, and population density.

Results: In adjusted models, the HR for the highest compared with the lowest quartile of each metal ranged from 0.78 (95% CI: 0.59, 1.04) for chromium to 1.33 (95% CI: 0.98, 1.79) for mercury.

Conclusions: Overall, we found limited evidence for the association between adulthood ambient exposure to metals and risk of PD. The results for mercury need to be confirmed in future studies.

Citation: Palacios N, Fitzgerald K, Roberts AL, Hart JE, Weisskopf MG, Schwarzschild MA, Ascherio A, Laden F. 2014. A prospective analysis of airborne metal exposures and risk of Parkinson disease in the Nurses’ Health Study Cohort. Environ Health Perspect 122:933–938; http://dx.doi.org/10.1289/ehp.1307218

## Introduction

Parkinson disease (PD) is the second most prevalent neurodegenerative disorder, after Alzheimer disease (Lang and Lozano 1998). Exposure to metals has been implicated in the pathogenesis of PD. Manganese intoxication is recognized as a cause of parkinsonism at high levels of exposure (Guilarte 2010; Jankovic 2005). However, the pathology of manganese intoxication is distinct from that of PD (Jankovic 2005), and the causal association of exposure to manganese with PD continues to be debated (Fored et al. 2006; Fryzek et al. 2005; Mortimer et al. 2012). For example, a study that compared the food habits of 250 patients and 388 controls found that a high manganese intake combined with a high intake of iron was significantly associated with PD (Powers et al. 2003). In another study in Quebec, Canada, a slightly higher although not statistically significant risk of PD was observed among participants with occupational exposure to manganese, iron, and aluminum (Zayed et al. 1990). At the same time, many studies of manganese and PD have been null (Hertzman et al. 1994; Seidler et al. 1996; Semchuk et al. 1993; Vieregge et al. 1995).

There has also been some evidence of onset of PD following occupational (Coon et al. 2006; Kuhn et al. 1998) as well as nonoccupational (Weisskopf et al. 2010) exposure to high levels of lead. Increased brain iron levels have been found in PD patients by some investigators, although this has not been confirmed in all studies (Logroscino et al. 1998, 2006, 2008). Some but not all studies have reported positive associations between PD and exposure to copper (e.g., Gorell et al. 1997). Furthermore, mercury measured in blood, urine, and hair has been positively associated with PD (Ngim and Devathasan 1989).

To our knowledge, to date only two epidemiologic studies have assessed exposure to airborne metals and PD in nonoccupational cohorts. A case–control study in Canada by Finkelstein and Jerrett (2007) reported a modest association between airborne manganese and PD. In a study of U.S. Medicare beneficiaries, Willis et al. (2010) used county-level data from the U.S. Environmental Protection Agency (EPA) Toxic Release Inventory (TRI) (U.S. EPA 2010b) on copper, lead, and manganese and found significant associations between residence in urban counties with high levels of release of manganese and PD.

In this study, we examined the association between census tract–level air emissions of antimony, arsenic, cadmium, chromium, lead, manganese, mercury, and nickel, and risk of PD in a large prospective cohort of female nurses.

## Methods

*Study population*. This study was conducted using data from the Nurses’ Health Study (NHS), an ongoing prospective cohort of female nurses initiated in 1976 and followed with biennial questionnaires collecting residential location and information on lifestyle factors and health outcomes. Residential locations were available throughout follow-up, which corresponds to exposure during adulthood in this cohort. At the initiation of the cohort in 1976, the 121,701 study participants were between 30 and 55 years of age and resided in 11 states (California, Connecticut, Florida, Maryland, Massachusetts, Michigan, New York, New Jersey, Pennsylvania, Ohio, and Texas). For each follow-up cycle, the rate of follow up has been > 90%. Information on state, county, and census tract of residence was derived from the residential address updated every 2 years. Detailed description of the cohort is provided elsewhere (Colditz et al. 1997). Data on airborne metal exposures were available for 97,430 women at baseline in 1990.

*PD ascertainment*. A question regarding PD onset and diagnosis was first asked in 1994 and has been asked every 2 years since. The ascertainment method for PD in this study has been described in detail previously (Ascherio et al. 2001). Briefly, each study participant who reports PD is sent a written request for consent to contact her treating neurologist (or internist, if the neurologist is not available). Once consent is provided by the participant, the doctor is contacted for a copy of the medical record and asked to complete a questionnaire documenting the likelihood of the diagnosis of PD. The medical records are reviewed by a neurologist/movement disorder specialist (M.A.S.) who is blinded to the exposure status of the participant. We considered confirmed cases to be participants with medical record evidence of a final diagnosis of PD by a treating neurologist, or medical record evidence of at least two cardinal signs of PD (bradykinesia, rigidity, or rest tremor) in the absence of information suggesting an alternate diagnosis. Women who self-reported a PD diagnosis before 1990 or had evidence in their medical record indicating onset before 1990 were excluded from the study.

*Airborne metals exposure ascertainment*. We used data on antimony, arsenic, cadmium, chromium, lead, manganese, mercury, and nickel exposure from the National Air Toxics Assessments (NATA) (U.S. EPA 2011). NATA includes data on emissions of hazardous air pollutants (HAPs) from a variety of sources including major stationary sources (such as factories), other sources (such as dry cleaners, small manufacturers, and wildfires), and traffic sources (cars, boats). The U.S. EPA created this inventory by drawing on data from state and local air pollution inventories and, if those were not available, on existing databases related to the U.S. EPA’s air toxics regulatory program, followed by the U.S. EPA TRI (U.S. EPA 2010b). NATA uses a complex dispersion model, ASPEN (Assessment System for Population Exposure Nationwide) (U.S. EPA 2010a), which estimates annual average concentrations of the HAPs for each census tract in the contiguous United States and Puerto Rico. The model incorporates information about the rate, location, and height of release; meteorological factors; and pollutant-specific factors such as radioactive decay, deposition, and secondary formation. HAP data were downloaded from the U.S. EPA website on 23 June 2010, and additional archived data were received on compact disc from the U.S. EPA (2011). HAPs data from 1990, 1996, and 1999 were available. We linked the HAP data with the NHS, using U.S. census state, county and tract identifiers (U.S. Census Bureau 2010). We used updated metal exposure in our analyses: metal values measured in 1990 were assigned for cases with onset prior to 1996, metal measures in 1996 were assigned for cases with onset between 1996 and 2000, and metal measures in 1999 were assigned for cases with onset after 2000. We estimated associations between PD and the following metals available in all years: antimony, arsenic, cadmium, chromium, lead, manganese, mercury, and nickel. Although the U.S. EPA specifically advises against combining metal concentrations measured at different time periods (U.S. EPA 2011), we performed a sensitivity analysis of associations with estimates of cumulative airborne metal exposures based on updated measurements at each time period, which is of interest because of the long preclinical phase of PD.

*Statistical analyses*. We used Cox proportional hazards models adjusted for age in months (crude model), as well as a multivariable model adjusted additionally for smoking (one variable defined as never/past/current and another continuous pack-years variable at baseline) and census tract–level population density (calculated as the number of people in the tract divided by the square miles of the tract, in quartiles) to calculate hazard ratios (HRs) for the association between exposure to airborne metals and risk of PD. We also conducted sensitivity analyses further adjusting for tract-level income (quartiles). Person-years of follow-up were calculated from baseline in 1990, through the end of follow-up (30 June 2008), death, or date of PD onset, whichever occurred earlier. The relationship between PD onset and metals exposure was examined for each metal individually, coded in quartiles (using cutoffs based on the exposure distribution over the entire study period), or continuously in separate models. Because smoking has been established as protective against PD based in multiple epidemiologic studies (Hernán et al. 2001), we performed additional analyses stratified by smoking status at baseline (ever vs. never smoker) and tested for interaction between each of the metals and smoking, by using the likelihood ratio test to compare a model that included a product term between smoking (ever/never) and the metal coded as an ordinal variable to a model without such a term. We also conducted additional analyses stratified by population density in 1990—the time of the metal exposure assessment—to examine the potential interaction of rural versus urban living with the effects of airborne metals. In these analyses, women residing in counties with ≥ 250,000 inhabitants were considered urban dwellers, whereas those in counties with < 250,000 inhabitants were considered rural dwellers.

All analyses were performed using SAS version 9.1 (SAS Institute Inc., Cary, NC). All analyses were conducted at the 0.05 alpha level, and all tests were two-sided. *p*-Values for trend test were based on a linear model through the quartile medians. All members of the NHS provide informed consent, implied through the return of the questionnaires. The study was approved by the institutional review of Brigham and Women’s Hospital.

## Results

Between the study baseline in 1990 and the end of the study in 2008, we confirmed 425 cases of PD with data available on metal exposures. Table 1 shows the baseline characteristics of the study participants. Age, body mass index (BMI), and smoking did not differ with quartile of total metal exposure (constructed as the sum of all metals in the study). Participants residing in census tracts with the lowest quartile of metal exposure also lived in tracts with the lowest median family income and lowest population density. Metal exposures were highly intercorrelated (Table 2), with Spearman correlation coefficients ranging from 0.38 to 0.68.

**Table 1:** Age-standardized characteristics at study baseline in 1990 of the 97,430 female NHS participants by quartile of total metal exposure (mean ± SD or percentage).

Characteristic	Quartile 1	Quartile 2	Quartile 3	Quartile 4
Age (years)	57.8 ± 7.2	57.6 ± 7.2	57.7 ± 7.1	58.0 ± 7.1
Pack-years smoking	11.8 ± 18.1	12.4 ± 18.5	12.0 ± 18.4	11.3 ± 18.0
BMI	25.9 ± 4.9	25.8 ± 4.9	25.9 ± 4.9	25.9 ± 5.0
Never-smoker (%)	45	42	43	44
Median tract household income, 1990	55,211 ± 9,501	66,534 ± 25,586	68,866 ± 27,274	66,096 ± 27,507
Median tract population density, 1990 (average persons/square mile)	1,450 ± 3,840	2,783 ± 4,458	4,010 ± 6,770	8,008 ± 15,744
Urban dwelling (% living in county with ≥ 250,000 inhabitants)	30	70	80	80

**Table 2:** Spearman correlations between the metals examined in this study.

Metal	Antimony	Arsenic	Cadmium	Chromium	Lead	Manganese	Mercury	Nickel
Antimony	1.00	0.57	0.52	0.38	0.54	0.54	0.50	0.60
Arsenic		1.00	0.68	0.54	0.66	0.61	0.61	0.62
Cadmium			1.00	0.51	0.64	0.59	0.66	0.58
Chromium				1.00	0.55	0.50	0.44	0.55
Lead					1.00	0.65	0.57	0.57
Manganese						1.00	0.47	0.52
Mercury							1.00	0.58
Nickel								1.00

Metals exposures were not significantly associated with PD (Table 3) in age-adjusted or multivariable (age, smoking, and population density)–adjusted models. In the main analyses, there was a suggestion of a positive monotonic association with exposure to mercury [the HR comparing to the top quartile of mercury exposure with the bottom quartile was 1.33 (95% CI: 0.98, 1.79; *p*_trend_ = 0.10)].

**Table 3:** Exposure to individual metal HAPs^*a*^ and risk of PD among participants on the NHS (*n* = 97,430) follow-up, 1990–2008, by quartile (Q) of each metal exposure.

Metal HAP	Median concentration (μg/m^3^)	Person-years	Cases	HR (95% CI)		*p*_trend_^*c*^
				Age adjusted	Fully adjusted^*b*^	
Antimony
Q1	0.000034	46,3350	113	1.00 (Referent)	1.00 (Referent)
Q2	0.000138	45,0715	104	0.97 (0.74, 1.27)	0.98 (0.75, 1.28)
Q3	0.000287	44,4776	104	1.00 (0.76, 1.30)	1.01 (0.77, 1.33)
Q4	0.000682	42,6213	104	1.01 (0.77, 1.32)	1.04 (0.78, 1.38)	0.70
Arsenic
Q1	0.000073	45,7965	117	1.00 (Referent)	1.00 (Referent)
Q2	0.000173	49,4663	94	0.84 (0.64, 1.10)	0.86 (0.65, 1.13)
Q3	0.000293	44,7912	112	1.01 (0.78, 1.30)	1.03 (0.78, 1.37)
Q4	0.000610	42,4514	102	0.94 (0.72, 1.23)	0.95 (0.71, 1.27)	0.95
Cadmium
Q1	0.000025	44,3659	120	1.00 (Referent)	1.00 (Referent)
Q2	0.000097	44,2193	95	1.06 (0.82, 1.38)	1.08 (0.83, 1.42)
Q3	0.000204	44,8139	116	1.02 (0.78, 1.32)	1.04 (0.79, 1.38)
Q4	0.000474	45,1063	94	0.90 (0.68, 1.19)	0.90 (0.67, 1.22)	0.26
Chromium
Q1	0.000165	44,3659	120	1.00 (Referent)	1.00 (Referent)
Q2	0.000478	44,2193	95	0.86 (0.67, 1.10)	0.86 (0.67, 1.11)
Q3	0.000926	44,8139	116	1.05 (0.83, 1.33)	1.05 (0.82, 1.34)
Q4	0.001961	45,1063	94	0.80 (0.61, 1.03)	0.78 (0.59, 1.04)	0.11
Lead
Q1	0.001971	45,9922	117	1.00 (Referent)	1.00 (Referent)
Q2	0.002896	45,2125	100	0.91 (0.70, 1.19)	0.92 (0.70, 1.22)
Q3	0.004890	44,2968	108	0.99 (0.76, 1.28)	0.99 (0.75, 1.31)
Q4	0.010354	43,0039	100	0.91 (0.70, 1.19)	0.90 (0.67, 1.22)	0.54
Manganese
Q1	0.001109	45,9870	101	1.00 (Referent)	1.00 (Referent)
Q2	0.002488	45,7074	128	1.30 (1.00, 1.68)	1.30 (1.00, 1.70)
Q3	0.004118	44,2783	96	0.99 (0.75, 1.32)	1.01 (0.75, 1.35)
Q4	0.007797	42,5327	100	1.05 (0.79, 1.38)	1.04 (0.77, 1.40)	0.58
Mercury
Q1	0.001543	44,9057	96	1.00 (Referent)	1.00 (Referent)
Q2	0.001649	45,4765	106	1.14 (0.87, 1.50)	1.15 (0.87, 1.52)
Q3	0.001867	45,7804	111	1.20 (0.92, 1.58)	1.24 (0.93, 1.65)
Q4	0.002405	42,3428	112	1.28 (0.97, 1.68)	1.33 (0.99, 1.79)	0.10
Nickel
Q1	0.000873	45,6523	109	1.00 (Referent)	1.00 (Referent)
Q2	0.002485	44,8290	118	1.15 (0.88, 1.48)	1.02 (0.76, 1.34)
Q3	0.004934	45,1511	107	1.02 (0.78, 1.33)	0.91 (0.67, 1.24)
Q4	0.011718	42,8731	91	0.91 (0.68, 1.20)	1.01 (0.79, 1.24)	0.25
^***a***^HAP metal levels were obtained from the U.S. EPA (2011). We used updated metal exposure incorporating all years of HAP measurement in our analyses. ^***b***^Adjusted for age and smoking (never, past, current, and pack-years) and population density (quartiles). ^***c***^Based on linear model through the quartile medians.						

Because at the census tract level total metal exposure was correlated with income (Table 1), we conducted additional sensitivity analyses adjusted for income. This adjustment did not substantially influence the results (data not shown). The results of sensitivity analyses that used cumulative updating were not significantly different from our primary analysis: Mercury was still the only metal that gave a suggestion of an association with PD risk (*p*_trend_ = 0.14) in these analyses (data not shown).

In analyses stratified by smoking (Table 4), we did not observe that smoking had any statistically significant effect modification of associations with any of the metals. Among never-smokers, we observed a significant increasing risk of PD with higher mercury exposure (HR comparing top with bottom quartile: 1.68; 95% CI: 1.11, 1.25; *p*_trend_ = 0.04) but not among ever-smokers (HR:0.99; 95% CI: 0.63, 1.55; *p*-trend = 0.85); the *p*-value for interaction with smoking was not significant (*p*_interaction_ = 0.29). We did not observe evidence of interactions between smoking and any of the other metals in the study.

**Table 4:** Exposure to individual metal HAPs^*a*^ and risk of PD among participants on the NHS (*n* = 97,430) follow-up, 1990–2008, by quartile (Q) of each metal exposure stratified by smoking status.

Metal HAP	Never-smoker				Ever-smoker				*p*_interaction_
	Person-years	Cases	HR (95% CI)^*b*^	*p*_trend_^*c*^	Person years	Cases	HR (95% CI)^*b*^	*p*_trend_^*c*^	
Antimony
Q1	205,420	60	1.00 (Referent)		249,179	53	1.00 (Referent)
Q2	198,308	61	1.06 (0.74, 1.52)		241,434	43	0.88 (0.59, 1.33)
Q3	193,544	51	0.90 (0.61, 1.32)		236,848	53	1.15 (0.78, 1.71)
Q4	184,744	55	0.99 (0.67, 1.47)	0.95	217,866	48	1.10 (0.73, 1.67)	0.48	0.58
Arsenic
Q1	210,463	63	1.00 (Referent)		237,236	48	1.00 (Referent)
Q2	194,833	48	0.83 (0.56, 1.23)		245,601	42	0.88 (0.58, 1.32)
Q3	186,002	60	1.08 (0.73, 1.59)		244,303	42	0.94 (0.61, 1.43)
Q4	190,718	56	0.96 (0.64, 1.43)	0.94	218,188	51	0.92 (0.59, 1.42)	0.90	0.97
Cadmium
Q1	209,521	60	1.00 (Referent)		234,166	51	1.00 (Referent)
Q2	957,711	59	1.06 (0.73, 1.54)		244,381	54	1.08 (0.73, 1.62)
Q3	185,869	53	0.99 (0.66, 1.48)		251,031	54	1.04 (0.68, 1.59)
Q4	190,854	55	0.97 (0.66, 1.46)	0.84	215,750	38	0.80 (0.50, 1.27)	0.18	0.46
Chromium
Q1	202,837	65	1.00 (Referent)		229,154	55	1.00 (Referent)
Q2	189,959	50	0.86 (0.61, 1.23)		238,065	44	0.82 (0.56, 1.19)
Q3	190,332	61	1.10 (0.78, 1.55)		242,816	55	0.97 (0.66, 1.40)
Q4	198,888	51	0.81 (0.55, 1.20)	0.33	235,293	43	0.74 (0.49, 1.13)	0.24	0.92
Lead
Q1	208,472	64	1.00 (Referent)		239,836	53	1.00 (Referent)
Q2	190,940	47	0.81 (0.55, 1.20)		248,120	53	1.02 (0.68, 1.54)
Q3	189,998	61	1.01 (0.69, 1.50)		238,962	46	0.92 (0.59, 1.42)
Q4	192,605	55	0.81 (0.54, 1.20)	0.90	218,411	45	0.92 (0.58, 1.44)	0.63	0.86
Manganese
Q1	198,449	52	1.00 (Referent)		252,400	49	1.00 (Referent)
Q2	197,675	73	1.37 (0.95, 1.96)		247,611	54	1.19 (0.80, 1.77)
Q3	192,392	51	0.96 (0.64, 1.44)		232,493	45	1.08 (0.71, 1.65)
Q4	193,500	51	0.92 (0.61, 1.40)	0.98	212,824	49	1.20 (0.78, 1.84)	0.37	0.49
Mercury
Q1	206,460	47	1.00 (Referent)		217,568	49	1.00 (Referent)
Q2	199,558	63	1.46 (0.99, 2.16)		245,796	43	0.84 (0.56, 1.29)
Q3	191,627	52	1.30 (0.85, 1.98)		255,953	58	1.10 (0.73, 1.67)
Q4	184,371	65	1.68 (1.11, 2.55)	0.04	226,011	47	0.99 (0.63, 1.55)	0.85	0.29
Nickel
Q1	211,260	57	1.00 (Referent)		234,868	52	1.00 (Referent)
Q2	195,949	63	1.21 (0.83, 1.76)		239,515	55	1.09 (0.74, 1.63)
Q3	191,980	59	1.13 (0.76, 1.68)		245,360	48	0.91 (0.59, 1.40)
Q4	182,828	48	0.96 (0.62, 1.47)	0.46	225,585	42	0.86 (0.55, 1.35)	0.36	0.90
^***a***^HAP metal levels were obtained from the U.S. EPA (2011). We used updated metal exposure incorporating all years of HAP measurement in our analyses. ^***b***^Adjusted for age and population density (quartiles). ^***c***^Based on linear model through the quartile medians.									

In analyses stratified by population density (Table 5), we observed a marginally significant interaction for arsenic (*p*_interaction_ = 0.06) consistent with evidence of a negative association among those living in less densely populated counties versus a weak positive association in highly populated counties. For most of the metals in the study, the relative risks among participants living in urban counties were higher than among those living in rural counties, although none of the other interaction tests were significant. The relative risk was particularly high for mercury exposure among those living in urban counties (HR comparing top quartile of exposure with bottom quartile was 1.84 (95% CI: 1.13, 2.99; *p*_trend_ = 0.14), although risk was also elevated in the low population density group (HR: 1.32; 95% CI: 0.79, 1.29) and the *p*-value did not indicate an interaction (*p*_interaction_ = 0.86).

**Table 5:** Exposure to individual metal HAPs^*a*^ and risk of PD among participants on the NHS (*n* = 97,430) follow-up, 1990–2008, by quartile (Q) of each metal exposure stratified by county-level population density low (< 250,000 persons per county) vs. high (≥ 250,000 persons per county).

Metal HAP	Low population density				High population density				
	Person-years	Cases	HR (95% CI)^*b*^	*p*_trend_^*c*^	Person-years	Cases	HR (95% CI)^*b*^	*p*_trend_^*c*^	*p*_interaction_
Antimony
Q1	256,829	68	1.00 (Referent)		206,521	45	1.00 (Referent)
Q2	179,049	61	1.13 (0.79, 1.64)		271,666	53	0.92 (0.62, 1.37)
Q3	113,333	26	0.93 (0.59, 1.48)		331,443	78	1.10 (0.76, 1.58)
Q4	64,700	14	0.88 (0.49, 1.59)	0.19	361,513	90	1.10 (0.77, 1.60)	0.35	0.21
Arsenic
Q1	307,615	90	1.00 (Referent)		150,351	27	1.00 (Referent)
Q2	150,741	35	0.84 (0.56, 1.26)		303,922	59	1.06 (0.67, 1.68)
Q3	80,920	20	0.91 (0.55, 1.51)		366,992	92	1.33 (0.85, 2.07)
Q4	74,637	14	0.65 (0.36, 1.15)	0.15	349,877	88	1.28 (0.81, 2.01)	0.37	0.06
Cadmium
Q1	305,054	84	1.00 (Referent)		149,459	27	1.00 (Referent)
Q2	154,477	42	1.08 (0.74, 1.59)		298,153	71	1.27 (0.81, 1.99)
Q3	75,229	15	0.81 (0.46, 1.42)		377,375	93	1.26 (0.81, 1.96)
Q4	79,151	18	0.86 (0.51, 1.45)	0.45	346,155	75	1.03 (0.65, 1.64)	0.43	0.60
Chromium
Q1	298,746	86	1.00 (Referent)		144,913	34	1.00 (Referent)
Q2	161,051	38	0.87 (0.60, 1.27)		281,142	57	0.92 (0.64, 1.32)
Q3	84,838	23	0.99 (0.63, 1.55)		363,301	93	1.15 (0.82, 1.61)
Q4	69,277	12	0.63 (0.34, 1.17)	0.19	381,786	82	0.88 (0.61, 1.27)	0.35	0.31
Lead
Q1	319,326	90	1.00 (Referent)		140,596	27	1.00 (Referent)
Q2	148,960	38	1.02 (0.69, 1.50)		303,166	62	0.99 (0.62, 1.57)
Q3	92,101	21	0.90 (0.55, 1.47)		350,867	87	1.15 (0.73, 1.80)
Q4	53,525	10	0.71 (0.36, 1.38)	0.26	376,514	90	1.04 (0.65, 1.65)	0.84	0.17
Manganese
Q1	266,167	64	1.00 (Referent)		193,703	37	1.00 (Referent)
Q2	161,175	50	1.32 (0.90, 1.91)		295,899	78	1.36 (0.91, 2.02)
Q3	101,067	24	1.02 (0.63, 1.64)		341,716	72	1.05 (0.70, 1.58)
Q4	85,503	21	1.02 (0.61, 1.69)	0.61	339,824	79	1.08 (0.72, 1.63)	0.70	0.91
Mercury
Q1	285,651	75	1.00 (Referent)		163,406	21	1.00 (Referent)
Q2	174,527	41	0.99 (0.67, 1.46)		280,238	65	1.77 (1.08, 2.89)
Q3	92,534	23	1.09 (0.67, 1.77)		365,271	88	1.80 (1.11, 2.91)
Q4	61,201	20	1.32 (0.79, 2.19)	0.29	362,227	92	1.84 (1.13, 2.99)	0.14	0.86
Nickel
Q1	312,521	82	1.00 (Referent)		144,002	27	1.00 (Referent)
Q2	157,280	45	1.21 (0.82, 1.76)		291,010	73	1.27 (0.81, 1.98)
Q3	82,026	18	0.95 (0.56, 1.61)		369,485	89	1.14 (0.73, 1.78)
Q4	62,086	14	0.93 (0.52, 1.65)	0.66	366,645	77	1.00 (0.63, 1.58)	0.33	0.84
^***a***^HAP metal levels were obtained from the U.S. EPA (2011). We used updated metal exposure incorporating all years of HAP measurement in our analyses. ^***b***^Adjusted for age and smoking (never, past, current, and pack-years). ^***c***^Based on linear model through the quartile medians.									

## Discussion

In this prospective cohort study of female nurses, we did not observe a statistically significant association between U.S. EPA HAP-modeled concentrations overall and risk of PD. In adjusted models, the HR for the highest compared with the lowest quartiles of each metal ranged from 0.78 (95% CI: 0.59, 1.04) for chromium to 1.33 (95% CI: 0.98, 1.79) for mercury. The association with mercury was stronger in nonsmokers as well as among participants living in urban counties.

To our knowledge, to date only two studies have addressed ambient air pollution and risk of PD. In Hamilton, Ontario, Canada, in a case–control study designed to examine the association between traffic pollution in general and PD, Finkelstein and Jerrett (2007) observed a modest increase in risk of PD among individuals with higher exposure to airborne manganese. However, this study identified cases using prescription data from a drug registry or a physician diagnosis code from the Ontario Health Insurance Plan, resulting in potential inclusion of subjects with manganism and not true PD, and thus potentially augmenting the association seen for manganese. In contrast, our study relied on PD cases confirmed through neurologist medical record review.

In one study, Willis et al. (2010) used physician disease codes to identify over 35,000 incident PD cases in a database of 29 million Medicare beneficiaries of PD, and compared the risk of PD among participants living in urban counties with high versus low cumulative industrial release of copper, manganese, or lead based on GIS-derived estimates from the U.S. EPA TRI (U.S. EPA 2010b). A major advantage of that study was its large sample size. Willis et al. (2010) found that participants residing in counties with the highest 25% of manganese release had an almost 80% higher risk of PD compared with those living in the counties with the lowest 25% for lead, copper, and manganese release. However, this study relied only on direct emissions data from the U.S. EPA TRI as their exposure. As discussed in “Methods,” the U.S. EPA TRI data contribute to the NATA HAPs data used in our study; however, the NATA data are also supplemented by data from local air pollution inventories and other U.S. EPA air toxics databases (U.S. EPA 2011). The TRI are raw emissions data, whereas the NATA data used in our study (U.S. EPA 2011) include a dispersion model that accounts for dispersion of air pollution, including across tract and county lines. Thus, the NATA data should provide a more accurate measure of exposure to airborne metals than the TRI data. Also, the smallest geographic unit in the study by Willis et al. (2010) was county, whereas we were able to estimate pollution concentration estimates at the census tract level. In contrast to Willis et al., we did not observe an association between higher exposure to airborne manganese and risk of PD. Our primary analyses were not restricted to urban or rural areas, but we conducted additional analyses stratified by low versus high population density (dichotomized in the same way as by Willis et al., where counties with ≥ 250,000 inhabitants were considered urban). In our study, for most metals the observed HRs associated with metal exposure were higher in the high population–density strata than in the low population–density strata, although except for arsenic, for which we observed a marginally significant *p*_interaction_ of 0.06, none of the other tests for interaction were significant. Also, our study included only women, and all the participants were nurses. We cannot, therefore, exclude the possibility that our results would have been different had our study focused on men or on individuals occupationally exposed to pesticides or other chemicals. An interaction between manganese-containing fungicides and paraquat, for example, has been reported in animal models of PD (Thiruchelvam et al. 2000). Likewise, in humans, simultaneous exposure to maneb and paraquat in participants ≤ 60 years old was associated with a 4.17 odds of PD, whereas exposure to either pesticide alone was associated with a 2.27 odds of PD (Costello et al. 2009).

The association between exposure to mercury and PD in the present study is supported by some (Ngim and Devathasan 1989; Seidler et al. 1996) but not all (Semchuk et al. 1993; Wechsler et al. 1991) prior studies. The association with mercury was stronger among never-smokers and residents of urban counties (with ≥ 250,000 inhabitants). Mercury is a heavy metal, and could contribute to oxidative damage in the substantia nigra; however, other heavy metals, such as iron (Lezak 1995), could also have this effect, so it is unclear why we saw an association with PD with mercury but not other heavy metals in this study.

One limitation of our work is that the levels of airborne metals were not measured directly, but rather were based on linkage with the U.S. EPA HAP-modeled concentrations. The use of census tract–level modeled estimates of air pollution may have obscured a true association between airborne metals and PD. Additionally, PD is thought to have a long preclinical period, so the ideal measure of exposure would have been a cumulative lifetime exposure to airborne metals. However, only HAPSs measures in 1990, 1996, and 1999 were available, and according to the U.S. EPA (2011), it was not advisable to combine the data into cumulative analyses. Thus, in our primary analyses we used exposure from only one time point for each PD case, as appropriate. This could have potentially biased our results. However, we did conduct analyses combining the three separate metals assessments into a cumulative measure, and confirmed that these results did not differ from the results of our primary analyses (data not shown). Also, it is not known how much time the participants spent inside versus outside their homes, and the HAP data set is a measure of outdoor exposures only. Penetration of outdoor pollutants indoors is possible, but depends on the ventilation rates of the individual dwellings.

The strengths of this study include its large size and a long, prospective follow-up, which included a large number of PD cases confirmed through neurologist medical record review. The study area included the whole contiguous United States, allowing for a wide range of exposure values for the airborne metals of interest. Among the limitations, our study included only women who were unlikely to be occupationally exposed to metals, pesticides, or other toxicants that might interact with metals. However, this aspect of our study is also an advantage because this is one of the few studies of airborne metal exposure in a nonoccupational cohort.

## Conclusion

Overall, we found little evidence that airborne metal exposures were associated with PD in this large prospective cohort of female nurses. There was limited evidence of an association between mercury exposure and PD, particularly among never-smokers and among participants living in counties with populations ≥ 250,000 persons. The results suggest that exposure to airborne metals is by itself unlikely to be a major cause of PD among U.S. women without occupational exposures to metals. The lack of association with most metals in this study as well the observed association with mercury needs to be confirmed in future studies.

## References

[r1] Ascherio A, Zhang SM, Hernán MA, Kawachi I, Colditz GA, Speizer FE (2001). Prospective study of caffeine consumption and risk of Parkinson’s disease in men and women.. Ann Neurol.

[r2] Colditz GA, Manson JE, Hankinson SE (1997). The Nurses’ Health Study: 20-year contribution to the understanding of health among women.. J Womens Health.

[r3] Coon S Stark A Peterson E Gloi A Kortsha G Pounds J 2006 Whole-body lifetime occupational lead exposure and risk of Parkinson’s disease. Environ Health Perspect 114 1872 1876; 10.1289/ehp.9102 17185278 PMC1764163

[r4] Costello S, Cockburn M, Bronstein J, Zhang X, Ritz B (2009). Parkinson’s disease and residential exposure to maneb and paraquat from agricultural applications in the Central Valley of California.. Am J Epidemiol.

[r5] Finkelstein MM, Jerrett M (2007). A study of the relationships between Parkinson’s disease and markers of traffic-derived and environmental manganese air pollution in two Canadian cities.. Environ Res.

[r6] Fored CM, Fryzek JP, Brandt L, Nise G, Sjögren B, McLaughlin JK (2006). Parkinson’s disease and other basal ganglia or movement disorders in a large nationwide cohort of Swedish welders.. Occup Environ Med.

[r7] Fryzek JP, Hansen J, Cohen S, Bonde JP, Llambias MT, Kolstad HA (2005). A cohort study of Parkinson’s disease and other neurodegenerative disorders in Danish welders.. J Occup Environ Med.

[r8] Gorell JM, Johnson CC, Rybicki BA, Peterson EL, Kortsha GX, Brown GG (1997). Occupational exposures to metals as risk factors for Parkinson’s disease.. Neurology.

[r9] Guilarte TR 2010 Manganese and Parkinson’s disease: a critical review and new findings. Environ Health Perspect 118 1071 1080; 10.1289/ehp.0901748 20403794 PMC2920085

[r10] Hernán MA, Zhang SM, Rueda-deCastro AM, Colditz GA, Speizer FE, Ascherio A (2001). Cigarette smoking and the incidence of Parkinson’s disease in two prospective studies.. Ann Neurol.

[r11] Hertzman C, Wiens M, Snow B, Kelly S, Calne D (1994). A case-control study of Parkinson’s disease in a horticultural region of British Columbia.. Mov Disord.

[r12] Jankovic J (2005). Searching for a relationship between manganese and welding and Parkinson’s disease.. Neurology.

[r13] Kuhn W, Winkel R, Woitalla D, Meves S, Przuntek H, Müller T (1998). High prevalence of parkinsonism after occupational exposure to lead-sulfate batteries.. Neurology.

[r14] Lang AE, Lozano AM (1998). Parkinson’s disease.. N Engl J Med.

[r15] Lezak M. (1995). Neuropsychological Assessment..

[r16] Logroscino G, Chen H, Wing A, Ascherio A (2006). Blood donations, iron stores, and risk of Parkinson’s disease.. Mov Disord.

[r17] Logroscino G, Gao X, Chen H, Wing A, Ascherio A (2008). Dietary iron intake and risk of Parkinson’s disease.. Am J Epidemiol.

[r18] Logroscino G, Marder K, Graziano J, Freyer G, Slavkovich V, Lojacono N (1998). Dietary iron, animal fats, and risk of Parkinson’ s disease.. Mov Disord.

[r19] Mortimer JA, Borenstein AR, Nelson LM (2012). Associations of welding and manganese exposure with Parkinson disease: review and meta-analysis.. Neurology.

[r20] Ngim C, Devathasan G (1989). Epidemiologic study on the association between body burden mercury level and idiopathic Parkinson’s disease.. Neuroepidemiology.

[r21] Powers KM, Smith-Weller T, Franklin GM, Longstreth WT, Swanson PD, Checkoway H (2003). Parkinson’s disease risks associated with dietary iron, manganese, and other nutrient intakes.. Neurology.

[r22] Seidler A, Hellenbrand W, Robra B-P, Vieregge P, Nischan P, Joerg J (1996). Possible environmental, occupational, and other etiologic factors for Parkinson’s disease: a case-control study in Germany.. Neurology.

[r23] Semchuk KM, Love EJ, Lee RG (1993). Parkinson’s disease: a test of the multifactorial etiologic hypothesis.. Neurology.

[r24] Thiruchelvam M, Richfield EK, Baggs RB, Tank AW, Cory-Slechta DA (2000). The nigrostriatal dopaminergic system as a preferential target of repeated exposures to combined paraquat and maneb: implications for Parkinson’s disease.. J Neurosci.

[r25] U.S. Census Bureau. (2010). U.S. Census Bureau Homepage.. http://www.census.gov/.

[r26] U.S. EPA (U.S. Environmental Protection Agency). (2010a). The ASPEN Model.. http://www.epa.gov/ttn/atw/nata/aspen.html.

[r27] U.S. EPA (U.S. Environmental Protection Agency). (2010b). Toxics Release Inventory (TRI) Program Homepage.. http://www2.epa.gov/toxics-release-inventory-tri-program.

[r28] U.S. EPA (U.S. Environmental Protection Agency). (2011). Technology Transfer Network Air Toxics: National Air Toxics Assessments.. http://www.epa.gov/nata/.

[r29] Vieregge P, Heinzow B, Korf G, Teichert HM, Schleifenbaum P, Mosinger HU (1995). Long term exposure to manganese in rural well water has no neurological effects.. Can J Neurol Sci.

[r30] Wechsler LS, Checkoway H, Franklin GM, Costa LG (1991). A pilot study of occupational and environmental risk factors for Parkinson’s disease.. Neurotoxicology.

[r31] Weisskopf MG Weuve J Nie H Saint-Hilaire MH Sudarsky L Simon DK 2010 Association of cumulative lead exposure with Parkinson’s disease. Environ Health Perspect 118 1609 1613; 10.1289/ehp.1002339 20807691 PMC2974701

[r32] Willis AW, Evanoff BA, Lian M, Galarza A, Wegrzyn A, Schootman M (2010). Metal emissions and urban incident Parkinson disease: a community health study of Medicare beneficiaries by using geographic information systems.. Am J Epidemiol.

[r33] Zayed J Ducic S Campanella G Panisset J André P Masson H 1990 Facteurs environnementaux dans l’étiologie de la maladie de Parkinson [in French] Can J Neurol Sci 17 286 291 2207882

